# Anti-Invasive Peptide-Functionalized Nanotubes for Selective c‑Met Targeting and Metal Chelation

**DOI:** 10.1021/acsomega.6c03987

**Published:** 2026-07-08

**Authors:** Vincenzo Patamia, Noemi Ravaglia, Mariacristina Failla, Erika Saccullo, Elena Bruno, Vincenzo Abbate, Monica Montesi, Silvia Panseri, Giuseppe Floresta

**Affiliations:** † Department of Drug and Health Sciences, 9298 University of Catania, Viale Andrea Doria 6, 95125 Catania, Italy; ‡ Institute of Science, Technology and Sustainability for Ceramics (ISSMC), National Research Council of Italy, Via Granarolo 64, 48018 Faenza, Italy; § Department of Neuroscience, Imaging and Clinical Science, University of Studies “G. D’Annunzio”, 66100 Chieti, Italy; ∥ Department of Drug Science and Technology, University of Turin, Via Pietro Giuria 9, 10125 Turin, Italy; ⊥ Department of Physics and Astronomy “Ettore Majorana”, University of Catania, Via S. Sofia 64, 95123 Catania, Italy; # CNR-IMM, University of Catania, Via Santa Sofia 64, 95123 Catania, Italy; ∇ Department of Analytical, Environmental & Forensic Sciences, Faculty of Life Sciences & Medicine, King’s College London, London SE1 9NH, U.K.

## Abstract

This work describes the development of a sustainable and biocompatible drug delivery system using halloysite nanotubes (HNTs). The goal was to create a material that could both deliver the chemotherapy drug doxorubicin (DOX) and selectively target the tumor cells. The synthesis process was made environmentally friendly by using tetrahydropyran (Thp) as a solvent. The HNTs were first functionalized with an amino group using (3-aminopropyl)­triethoxysilane (APTES), followed by the conjugation of two different peptides, P1 and P2, via EDC coupling. Successful functionalization was confirmed by FTIR spectroscopy, and through thermogravimetric analysis (TGA), we determined the degree of functionalization (%*f*). The DOX loading efficiency was found to be 8.8% for all composites, indicating that the peptides did not interfere with drug loading. Drug release studies showed that HNT-NH-P1 exhibited a faster release rate, releasing 9% of DOX in 24 h, while HNT-NH-P2 showed a much slower release of only 3.5% over the same period. Furthermore, *in vitro* scratch assays revealed a selective antimigratory effect of HNT-NH-P1-DOX on c-Met-positive adenocarcinoma cells, suggesting a receptor-mediated targeting potential. Finally, as a feasibility proof-of-concept for potential radiopharmaceutical applications, the HNT-NH-P1 platform was successfully extended with a THP chelator, demonstrating a high coordination capacity for trivalent Ga. These results demonstrate the potential of HNTs as versatile platforms for targeted drug delivery and imaging applications.

## Graphical abstract

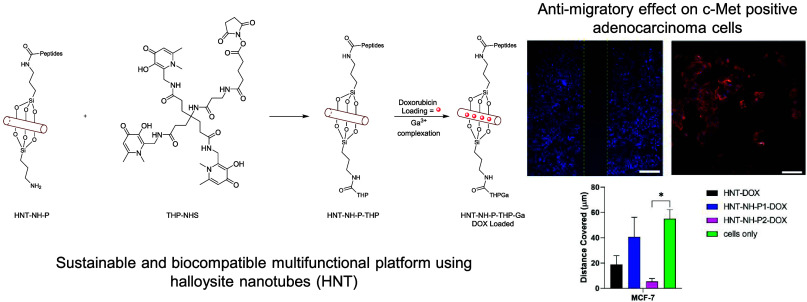

## 1 Introduction

The field of nanomedicine has rapidly advanced, offering innovative solutions for drug delivery with an improved efficacy and reduced side effects. The development of nanocarriers for targeted delivery has moved toward utilizing natural and biocompatible materials^1^ to minimize the risk of toxicity and immune responses. Beyond synthetic polymers, a wide range of naturally derived systems has been explored. For instance, polysaccharides like chitosan, hyaluronic acid, and alginic acid are widely used for their excellent biocompatibility, biodegradability, and ability to be easily modified.^2–4^ Lipid-based systems, such as liposomes, have become a cornerstone of nanomedicine due to their ability to encapsulate both hydrophilic and hydrophobic drugs.^5^ Similarly, protein-based nanoparticles, often derived from albumin or collagen, are attractive because of their inherent biocompatibility and specific biological recognition capabilities.^6^

Among these diverse natural nanomaterials, clay-based nanosystems have garnered significant attention due to their biocompatibility, high surface area, and cost effectiveness.^7^ Naturally occurring clay minerals like halloysite nanotubes (HNTs) are particularly promising because of their unique, hollow tubular structure and distinct inner and outer surface chemistries, which enable selective modification. HNTs are an aluminosilicate clay with a low toxicity profile, making them an excellent candidate for biomedical applications.^8–10^ Beyond drug delivery, these versatile nanoparticles have been widely explored across various fields. In catalysis, their high surface area and porous nature allow them to act as effective supports for catalysts.^11,12^ In sensing, HNTs can be used to create highly sensitive and selective sensors by incorporating active sensing elements within their lumen or on their surface.^13^ Their application in the medical field is particularly diverse, ranging from tissue-engineering scaffolds to controlled-release drug delivery systems.^14^

This work focuses on the development of a novel, environmentally friendly nanocarrier system using HNTs for the targeted delivery of the potent anticancer drug doxorubicin (DOX). The synthesis of this system incorporates principles of sustainable chemistry by substituting traditional tetrahydrofuran (THF) with tetrahydropyran (Thp), a more eco-friendly solvent, during the key 3-aminopropyl-triethoxysilane (APTES) functionalization step. The HNTs are specifically functionalized with two distinct peptides, enhancing their ability to bind to and carry the drug while also providing a targeting mechanism to improve the cellular uptake and therapeutic outcomes. This approach represents a balanced step toward efficient cancer therapeutics, leveraging the structural benefits of HNTs alongside targeted improvements in their synthetic sustainability.

## 2 Materials

### 2.1 General Information

All chemicals were purchased from Merck and VWR. THP-NHS (Tris­(hydroxypyridinone)) was synthesized as reported in the literature.^15^ Thin-layer chromatography (TLC) was performed using precoated aluminum-backed silica gel 60 F254 plates (Merck), and chromatograms were visualized under UV irradiation. Mass spectrometric analyses were conducted using either a Thermo Fisher LCQ DECA XP ion-trap mass spectrometer or a Waters Micromass ZQ single-quadrupole mass spectrometer. Analytical reversed-phase high-performance liquid chromatography (RP-HPLC) was carried out on an HP1050 HPLC system equipped with an autosampler, a quaternary pump, and a diode-array detector (DAD). Separations were performed on a Zorbax SB-C18 column (2.1 mm × 100 mm, 5 μm particle size) at a flow rate of 0.3 mL min^–1^. Detection was monitored over the wavelength range of 210–280 nm. Elution was achieved using a linear gradient of mobile phase B (acetonitrile containing 0.1% trifluoroacetic acid, TFA) in mobile phase A (water containing 0.1% TFA), increasing from 0 to 90% B over 20 min, with a total run time of 31 min.

### 2.2 Synthesis of Materials

#### 2.2.1 Synthesis of P1 and P2

The linear sequences [P1 = YLFSVHWPPLKA; P2 = QQTNWSL] were synthesized using a standard Fmoc solid-phase peptide synthesis (SPPS) approach. All Fmoc (9-fluorenylmethoxycarbonyl)-protected amino acids employed in this study carried the conventional acid-labile side-chain protecting groups. Peptide synthesis was performed on 2-chlorotrityl chloride resin (Novabiochem, loading capacity 1.47 mmol/g, 0.3 g), which served as the solid support. Prior to use, the resin was swollen in anhydrous dichloromethane (DCM, 3 mL) for 30 min at room temperature. For the loading of the resin 4 equiv. (equivalents) of the first Fmoc-protected amino acid, 8 equiv of DIPEA were dissolved in dry DCM (3 mL), and the reaction mixture was added to the resin. For peptide elongation, the reaction mixture was agitated on a tube roller mixer overnight at room temperature. The resin was subsequently washed three times with dichloromethane (DCM, 20 mL) and dimethylformamide (DMF, 20 mL), followed by capping of unreacted sites with methanol (MeOH). Peptide chain assembly was carried out manually using standard Fmoc-based solid-phase peptide synthesis (SPPS) protocols. Each coupling step employed 4 equiv of the appropriate Fmoc-protected amino acid, 4 equiv of Oxyma (ethyl cyanohydroxyiminoacetate), and 4 equiv of *N*,*N*′-diisopropylcarbodiimide (DIC) in anhydrous DMF (2 mL). Coupling reactions were allowed to proceed for 4 h at room temperature, after which Fmoc deprotection was achieved using 20% piperidine in DMF for 20 min. Upon completion of the synthesis, the peptides were cleaved from the resin using a cleavage cocktail consisting of trifluoroacetic acid (TFA)/phenol/water/triisopropylsilane (TIPS) (90:5:2.5:2.5, v/v/v/v). The cleavage mixture was stirred at room temperature for 1 h. The resulting solution was concentrated under reduced pressure, and the crude peptides were precipitated by addition to cold diethyl ether. The precipitated products were collected by centrifugation and dried under vacuum. Crude peptides were characterized by reverse-phase high-performance liquid chromatography (RP-HPLC) and mass spectrometry.

Final purification was performed by preparative RP-HPLC using a Gemini C18 AXIA column (100 mm × 30 mm, 5 μm, 110 Å). Mobile phase A consisted of water containing 0.1% TFA, while mobile phase B consisted of acetonitrile containing 0.1% TFA. Elution was carried out using a linear gradient from 0 to 40% mobile phase B over 60 min at a flow rate of 15 mL min^–1^. UV detection was performed at 281 nm. Collected fractions were analyzed by analytical HPLC-DAD, and those exhibiting the desired purity were pooled, lyophilized, and obtained as white solids. **P1** Calculated ESI^+^ [M + H]^+^: 1457.79, [M + 2H]^2+^: 729.40, [M + 3H]^3+^: 486.60. Measured [M + H]^+^: 1457.94, [M + 2H]^2+^: 729.98, [M + 3H]^3+^: 486.90 (see Figures S1 and S2). Calculated Elemental Analysis: C, 60.97; H, 7.19; N, 15.37; O, 16.46. Measured Elemental Analysis: C, 61.03; H, 7.14; N, 15.45; O, 16.38. **P2** Calculated ESI^+^ [M + H]^+^: 876.42. Measured ESI^+^ [M + H]^+^: 876.73 (see Figures S3 and S4). Calculated Elemental Analysis: C, 52.11; H, 6.56; N, 17.59; O, 23.74. Measured Elemental Analysis: C, 52.04; H, 6.59; N, 17.51; O, 23.86. See Figures S1–S4.

#### 2.2.2 Synthesis of HNT-NH_2_

A suspension of halloysite nanotubes (HNT, 400 mg) in tetrahydropyran (Thp, 10 mL) was treated with (3-aminopropyl)­triethoxysilane (APTES, 2 mL) and stirred at 65 °C for 12 h. Upon completion of the reaction, the mixture was centrifuged, and the resulting solid was collected and washed repeatedly with Thp (3 × 5 mL), with centrifugation after each washing step, to remove residual impurities. The purified material was then dried in an oven at 70 °C for 24 h, affording amino-functionalized halloysite nanotubes (HNT–NH_2_) as a solid (413 mg).

#### 2.2.3 Synthesis of HNT-NH-P1

Peptide P1 (2 mg) was suspended in tetrahydropyran (Thp, 4 mL) under a nitrogen atmosphere, and 1-ethyl-3-(3-(dimethylamino)­propyl)­carbodiimide (EDC, 1.2 mg) was added. The reaction mixture was stirred under nitrogen for 10 min to allow activation of the peptide carboxyl groups. Subsequently, HNT–NH_2_ (100 mg) was added, and the resulting suspension was stirred at room temperature under a nitrogen atmosphere for 24 h. Upon completion of the reaction, the mixture was centrifuged, and the resulting solid was collected and washed thoroughly with deionized water (3 × 5 mL) to remove unreacted reagents and byproducts. The purified material was then dried under reduced pressure to afford the peptide-functionalized halloysite nanotubes (HNT–NH–P1) as a solid (103 mg).

#### 2.2.4 Synthesis of HNT-NH-P2

Peptide P2 (1 mg) was suspended in tetrahydropyran (Thp, 4 mL) under a nitrogen atmosphere, and 1-ethyl-3-(3-(dimethylamino)­propyl)­carbodiimide (EDC, 1.0 mg) was added. The mixture was stirred under nitrogen for 10 min to allow activation of the carboxyl functionality. Subsequently, HNT–NH_2_ (100 mg) was introduced, and the resulting suspension was stirred at room temperature under a nitrogen atmosphere for 24 h. After completion of the reaction, the crude mixture was centrifuged, and the resulting solid was collected and washed with deionized water (3 × 5 mL) to remove residual reagents and byproducts. The purified material was then dried under vacuum, yielding peptide-functionalized halloysite nanotubes (HNT–NH–P2) as a solid (101 mg).

#### 2.2.5 Synthesis of HNT-NH-P1-THP

Seventeen mg of THP-NHS (MW: 980.04, 0.00173 mmol) were solubilized in 1 mL of Thp and left to stir for 15 min at room temperature. This solution was then added to 74 mg of HNT-NH-P1 already suspended in 3 mL of Thp and left to stir overnight at room temperature. The crude reaction mixture was then centrifuged and rinsed with Thp (3 × 3 mL) and then with H_2_O (3 × 3 mL) and then placed in an oven at 65 °C overnight to give 63 mg of HNT-NH-P1-THP.

#### 2.2.6 Synthesis of HNT-Ga

Ten mg of HNT and 10 mg of GaCl_3_ were suspended in 1 mL of deionized H_2_O and left to stir overnight at room temperature. The crude reaction mixture was then centrifuged and washed with H_2_O (3 × 1 mL) and left to dry in an oven at 65 °C overnight, yielding 7 mg of HNT-Ga.

#### 2.2.7 Synthesis of HNT-NH-P1-THP-Ga

Ten mg of HNT-NH-P1-THP and 10 mg of GaCl_3_ were suspended in 1 mL of deionized H_2_O and left to stir overnight at room temperature. The crude reaction mixture was then centrifuged and washed with H_2_O (3 × 1 mL) and left to dry in an oven at 65 °C overnight, yielding 8 mg of HNT-NH-P1-THP-Ga.

#### 2.2.8 Drug Loading and Release

To 3 mL of a 0.1 mg/mL DOX (Doxorubicin) solution in PBS, 3 mg of HNT-NH_2_ or HNT-NH-P1 or HNT-NH-P2 was added, i.e., with a DOX:HNTs ratio of 1:10 wt/wt, and left under agitation in the dark for 24 h. The DOX-loaded HNTs solutions were then centrifuged at 5000 rpm for 10 min and washed twice with PBS 1×. The supernatant was saved to measure the adsorption capacity of the composites using ultraviolet–visible (UV/vis, Jasco V-730 spectrophotometer and quartz cuvettes with an optical path length of 1 cm) spectroscopy. The loading efficiency was calculated as follows^16^

$$loading\;efficiency\;\left(\%\right)=\frac{weight\;of\;loaded\;DOX}{total\;weight\;of\;HNTs\;and\;loaded\;DOX}\times 100\%$$

The drug loading experiments for all functionalized composites were performed in triplicate (*n* = 3), yielding highly reproducible encapsulation efficiency values of 8.82 ± 0.15% for HNT-NH_2_, 8.78 ± 0.11% for HNT-NH-P1, and 8.81 ± 0.13% for HNT-NH-P2 (expressed as a rounded average of 8.8% in the text).

A total of 3 mg of the DOX-loaded composite was dispersed in 3 mL of phosphate-buffered saline (PBS, pH 7.4) or buffer solution at pH 5.5 under constant stirring at 37 °C in an Eppendorf tube. At predetermined time points, the suspension was centrifuged (5000 rpm, 2 min), and 2 mL of the supernatant was collected for quantification of released doxorubicin (DOX) using UV–Vis spectrophotometry. The DOX calibration curve, along with representative absorption spectra, is reported in Figure S5a,b.

### 2.3 Biological Experiment

HNT, HNT-DOX, HNT-NH_2_, HNT-NH-P1-DOX, and HNT-NH-P2-DOX samples were first dispersed in Milli-Q water at a concentration of 1 mg/mL. DOX (Merck, EP Reference Standard), dissolved at 1 mg/mL in Milli-Q water, was used as a positive control, whereas untreated cells served as the control group. For *in vitro* testing, the composites were subsequently diluted in cell culture medium to achieve concentrations corresponding to 2.5 and 5 μM DOX, based on the drug-loading results. For HNT samples without DOX, the same mass of nanocarrier as in the corresponding DOX-loaded samples was used to ensure equivalent carrier exposure considering the calculated drug-loading efficiency, i.e., 8.8% (88.0 μg/mg of HNT).

*In vitro* tests were performed to evaluate the biological activity of the synthesized composites toward two human breast adenocarcinoma cell lines: MCF-7 and MDA-MB-231 (ATCC HTB-22 and ATCC HTB26), which are a c-Met negative and c-Met positive cell lines, respectively. Cells were cultured in RPMI medium (Gibco) supplemented with 10% fetal bovine serum (FBS, Gibco) and 1% penicillin–streptomycin solution (100 U mL^–1^ and 100 μg mL^–1^, respectively; Gibco). Cultures were maintained at 37 °C in a humidified atmosphere containing 5% CO_2_. Cells were detached from culture flasks by trypsinization, collected by centrifugation, and resuspended for further use. Cell number and viability were determined using the trypan blue exclusion assay. All procedures were performed under a sterile biological laminar-flow hood. For cell viability and morphological analyses, MCF-7 and MDA-MB-231 cells were seeded at densities of 6.0 × 10^4^ and 3.0 × 10^4^ cells cm^–2^, respectively.

#### 2.3.1 Cell Viability Assessment

Quantitative evaluation of cell viability was carried out by Thiazolyl Blue Tetrazolium Bromide (MTT) after 72 h of incubation with the compounds, according to manufacturer’s instructions. Briefly, each sample was incubated with 10% (v/v) MTT solution (Merck, 5 mg/mL) in culture medium for 2 h at 37 °C, 5% CO_2_ atmosphere, and controlled humidity conditions. The metabolically active cells reacted with the tetrazolium salt in the MTT reagent to produce formazan crystals. Then, the culture medium was replaced by 200 μL of dimethyl sulfoxide (DMSO) to dissolve the formazan crystals derived from MTT conversion. After 15 min of incubation, the supernatants were analyzed using a UV–visible spectrophotometer (Multiskan FC, Thermo Scientific) by measuring absorbance at 570 nm (*n* = 3).

#### 2.3.2 Cell Morphology Evaluation

The morphology of MCF-7 and MDA-MB-231 cells was visualized by fluorescent staining of actin filaments. After 72 h of culture with HNT, HNT-DOX, HNT-NH_2_, HNT-NH-P1-DOX, and HNT-NH-P2-DOX at 2.5 μM and 5 μM, samples were fixed with 4% (w/v) paraformaldehyde (PFA, Merck KGaA) for 15 min, followed by cell membrane permeabilisation with 0.1% (v/v) Triton X-100 in PBS 1× for 15 min at room temperature. Actin filaments were stained using Actin Red 555 ReadyProbes Reagent (Invitrogen) for 30 min, and cell nuclei were counterstained with DAPI (600 nM, Invitrogen) in PBS 1× for 7 min. Fluorescent images were acquired using a Nikon Inverted Ti-E fluorescence microscope. One sample per group was analyzed (*n* = 1).

#### 2.3.3 Migration Assay

Cell migration ability was analyzed by applying an optimized model of the scratch assay.^17^ MCF-7 and MDA-MB-231 were seeded in a 24-well plate at a density of 4.0 × 10^4^ and 2.0 × 10^4^ cells/cm^2^, respectively.

After 72 h, the cell monolayer was scraped in a straight line to create a “scratch” with a p200 pipet tip; then, cells were washed with cell medium to remove cell debris, and HNT-DOX, HNT-NH-P1-DOX, and HNT-NH-P2-DOX were added at a 5 μM concentration, diluted in cell medium supplemented with only 2% FBS to slow down cell proliferation and better visualize cell migration over time. A first image of the scratch was acquired at time 0, then 72 h of incubation with the composites by a Nikon Inverted Ti-E Fluorescent microscope. For each captured image, five measurements of the scratch width were taken and quantitatively analyzed using ImageJ software. In addition, cells were fixed with 4% (w/v) paraformaldehyde (PFA), and cell nuclei were stained with DAPI prior to imaging. Fluorescence images were acquired using a fluorescence microscope. All experiments were performed in biological duplicate (*n* = 2) for each condition.

## 3 Statistical Analysis

All the results were plotted as mean ± standard error of the mean (SEM), and statistical analyses were performed by GraphPad Prism Software (Version 8.0). Cell viability data were analyzed by Two-way analysis of variance (Two-way ANOVA), followed by Dunnett’s Multiple Comparisons test. Cell migration data were analyzed by One-way ANOVA Dunnett’s Multiple Comparisons test. Statistically significant differences are reported in the graphs: *p value ≤ 0.05, **p value ≤ 0.01, ***p value ≤ 0.001, and ****p value ≤ 0.0001.

### 3.1 Characterization

#### 3.1.1 Infrared Spectroscopy

FTIR-ATR analyses were conducted using an FTIR Agilent Cary 630 equipped with an ATR sampling module. Thin films of the samples were applied to the ATR crystal and pressed gently. The results were derived from 512 scans acquired in the 4000–500 cm^–1^ range with a resolution of 2 cm^–1^ at room temperature.

#### 3.1.2 Thermogravimetric Analysis

Thermal gravimetric analysis (TGA) was used to study the thermal behavior under 1 atm of prepurified nitrogen, with a heating rate of 10 °C/min, in the temperature range of 50–900 °C.

#### 3.1.3 SEM-EDX

The synthesized material’s morphology was analyzed by scanning electron microscopy by using a Dual Beam Focused ion beam Versa 3D LoVac DualBeam in secondary electron mode using a 5 keV electron beam. The samples were also in situ analyzed by energy dispersive X-ray spectroscopy (EDX) using a 20 keV electron beam. Before analysis, all samples were sputtered with 5 nm of Au to ensure proper conductivity during measurements.

## 4 Results and Discussion

### 4.1 Synthesis

This work aims to use a sustainable and eco-biocompatible nanomaterial, based on the ability of halloysite nanotubes (HNTs) to deliver a drug such as doxorubicin, and at the same time, thanks to functionalization with specific peptides (1 and 2), to recognize and localize c-Met overexpressing tumor cells.^18^ The entire synthetic process is made even more sustainable thanks to the use of environmentally friendly solvents such as tetrahydropyran (Thp), used for the preparation of the nanocomposite (Scheme 1). The synthesis begins with the insertion of an amino termination using (3-aminopropyl)­triethoxysilane (APTES) and hydroxyl groups on the surface of the HNT, using Thp as a solvent. We then decided to conjugate the two peptides (P1 and P2, two peptides already reported to specifically bind to the c-Met receptor)^19–21^ using a coupling agent such as EDC, again using Thp as the solvent, exploiting the free carboxyl function of the peptides. Finally, we loaded the two systems obtained, HNT-NH-P1 and HNT-NH-P2, with DOX.

**Scheme 1:**
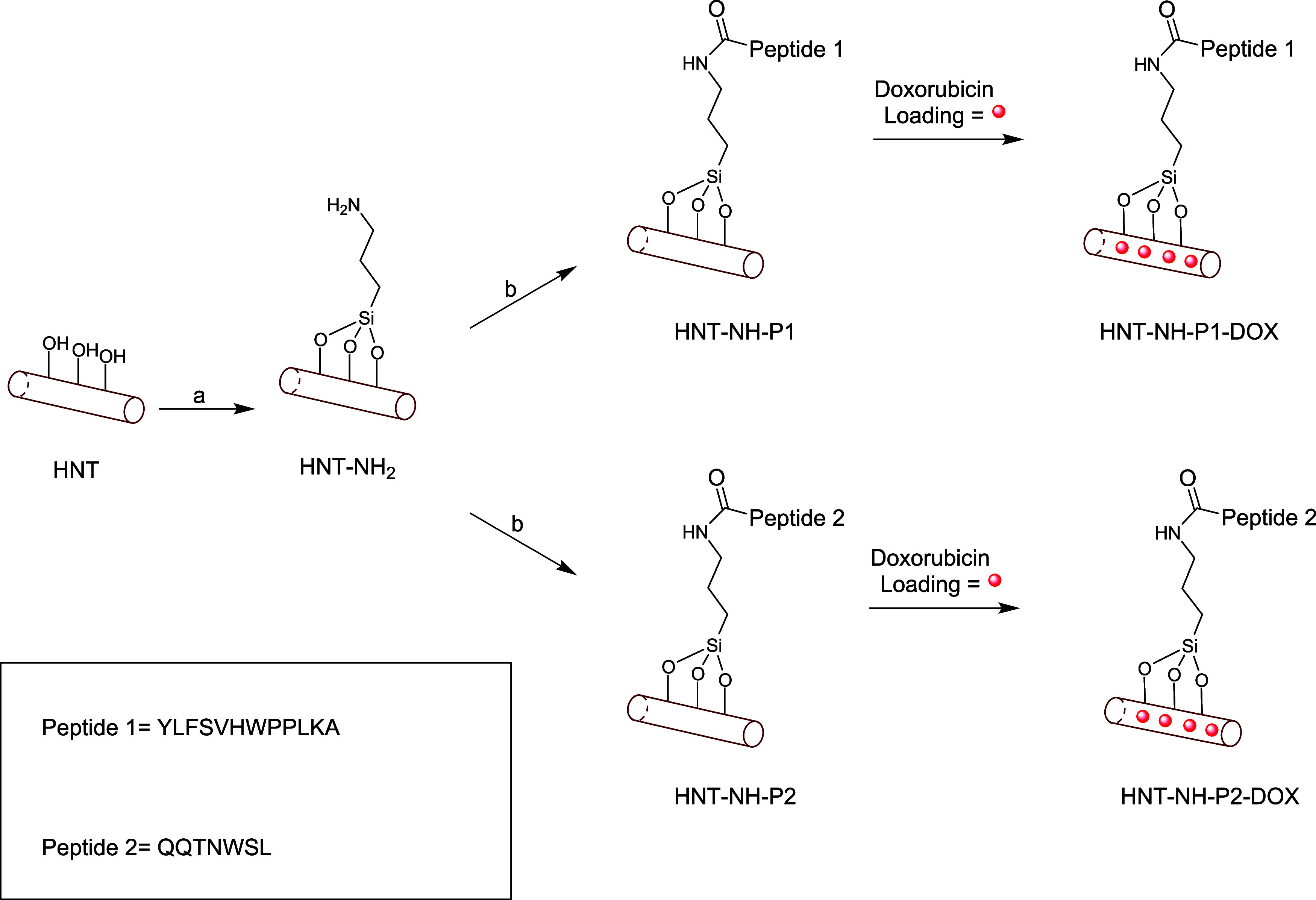
Preparation Scheme of HNT-NH-P1/P2 Loaded with Doxorubicin; (a) APTES, Thp, 65 °C, 12 h; (b) EDC, Thp, r.t., 24 h

### 4.2 Characterizations

Successful functionalization was confirmed by FTIR analysis. Figure 1 shows the FTIR spectra of HNT, HNT-NH_2_, HNT-NH-P1, and HNT-NH-P2, demonstrating successful functionalization. The bands associated with OH groups are evident in the HNT spectrum (black line): the Al–O–OH vibration is responsible for the peak at 907 cm^–1^, while the stretching vibrations of the Al–OH groups are responsible for the bands at 3696 and 3624 cm^–1^. In addition, a prominent O–Si–O peak is visible at approximately 1000 cm^–1^, while the apical Si–O stretching mode is responsible for the peak at 749 cm^–1^.^22,23^ Alongside these, there are signals relating to APTES functionalization (red line), namely the vibrational bands at 1500–1551 cm^–1^ attributed to N–H deformation and at 1450–1375 cm^–1^ due to the bending of C–H bonds in the carbon chain.^24^ The blue line and purple line in Figure 1 show the spectrum of the HNT-NH-P1 and HNT-NH-P2 composites, respectively, in which it is possible to observe both the signals mentioned above in the 1500–1375 cm^–1^ range and new signals in the 3000–2840 cm^–1^ region relating to the stretching of C–H bonds typical of the peptide chain.^24,25^

**Figure 1:**
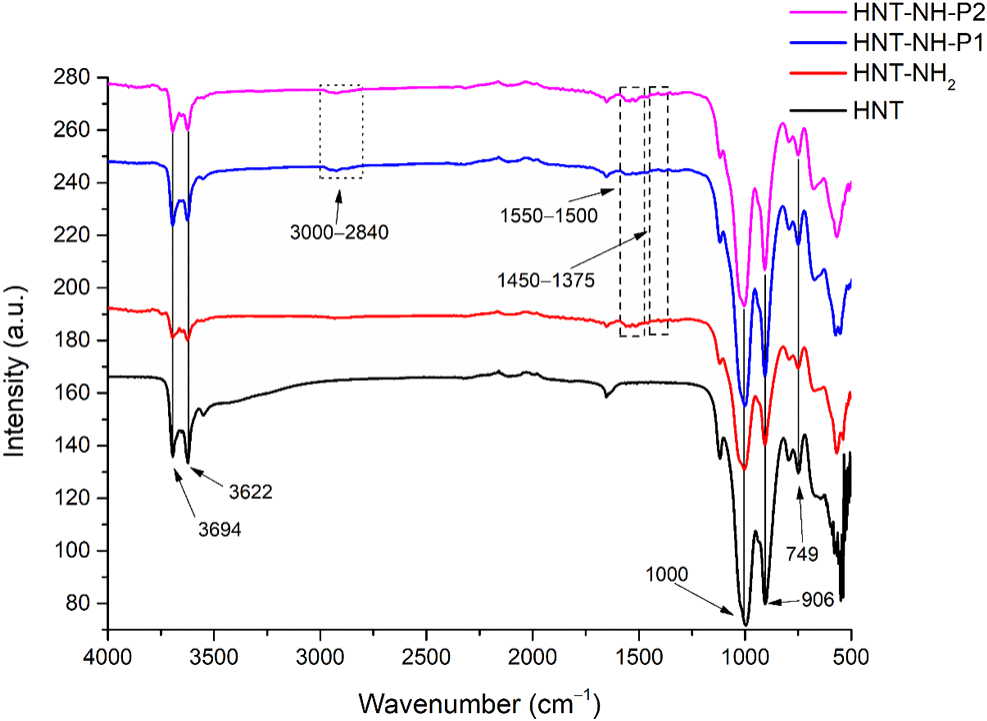
Stacked FTIR spectra of HNT (black line), HNT-NH_2_ (red line), HNT-NH-P1 (blue line), and HNT-NH-P2 (purple line).

In order to verify the thermal properties and degree of functionalization (%*f*) of the synthesized composites, thermogravimetric analyses were performed.^11,12^ The overlapping thermograms of the various composites HNT-NH_2_ (red line), HNT-NH-P1 (blue line), HNT-NH-P2 (purple line), and pristine HNT (black line) are shown in Figure 2.

**Figure 2:**
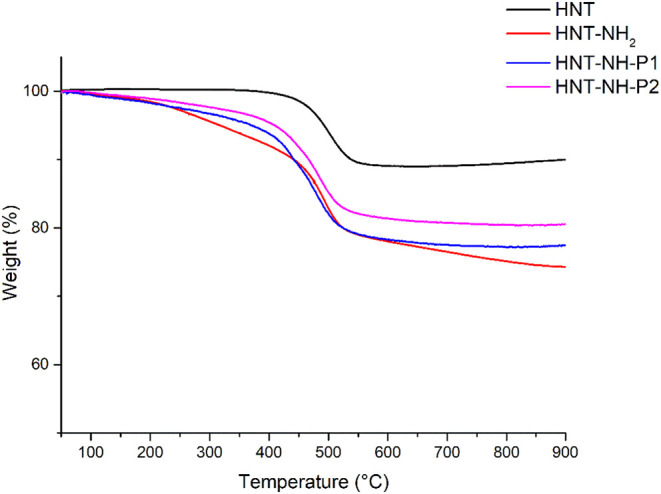
Thermogravimetric curves of HNT (black line), HNT-NH_2_ (red line), HNT-NH-P1 (blue line), and HNT-NH-P2 (purple line).

Table 1 shows the weight loss percentages of pristine HNT, HNT-NH_2_, HNT-NH-P1, and HNT-NH-P2. The degree of functionalization (%*f*) for the composite was calculated as reported in literature^11,12^ by considering the mass loss between 150 and 550 °C. In particular, we note that %*f* for HNT-NH_2_ is approximately 6%, HNT-NH-P1 is approximately 5.8%, and HNT-NH-P2 is approximately 3.2%. This difference in weight percentage is directly related to the intrinsic molecular weight of the single sequences (1457.79 g/mol for P1 vs 876.42 g/mol for P2). When converted to molar loading, both functionalization pathways yielded a comparable grafting density of approximately 0.04 mmol/g.

**Table 1:** Mass Loss Percentages of Pristine HNT, HNT-NH_2_, HNT-NH-P1, and HNT-NH-P2

sample	*T* < 150 °C	150 °C < *T* < 350 °C	350 °C < *T* < 550 °C	550 °C < *T* < 900 °C	residue%
HNT	1.1	1.8	12.3	1.1	83.7
HNT-NH_2_	0.95	5.37	14.74	4.57	74.3
HNT-NH-P1	1.15	3.35	16.50	1.46	77.54
HNT-NH-P2	0.46	2.56	14.76	1.45	80.77

Figure 3 shows SEM images of HNT-NH-P1-DOX (A–C) and HNT-NH-P2-DOX (D-F) at different magnifications. The images show that both composites have the same morphology typical of nanotubes.^12,23^ At higher magnifications (Figure 3C,F), the nanotube surfaces exhibit a rough, slightly modified surface texture. While this topographic roughness cannot be uniquely distinguished from the natural texture of raw HNTs by SEM analysis alone, the presence and uniform distribution of the organic peptide shell and the DOX payload are fully confirmed by the EDX elemental mapping.

**Figure 3:**
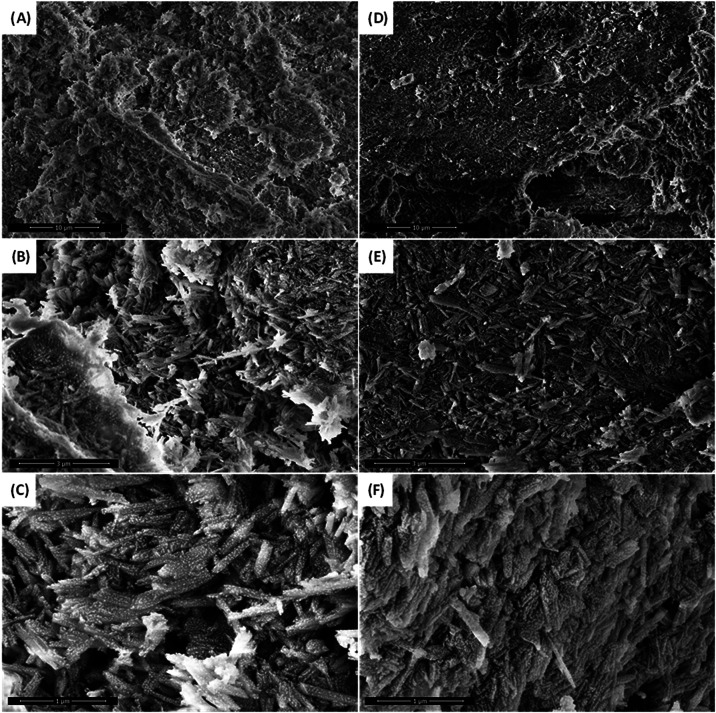
SEM micrographs of HNT-NH-P1-DOX (A–C) HNT-NH-P2-DOX (D–F).

This analysis confirms the presence of elements associated with both the inorganic matrix (Si, Al, and O) and the organic components derived from the peptides and DOX (C and N). The EDX maps of HNT-NH-P1-DOX and HNT-NH-P2-DOX are shown in Figures S6 and S7 of the Supporting Information, respectively.

The composites examined for DOX loading, HNT-NH_2_, HNT-NH-P1, and HNT-NH-P2, showed equal drug-loading efficiency, i.e., 8.8%. This value corresponds to 88.0 μg/mg of HNT, a payload that is significantly higher than that reported for DNA-wrapped HNTs by Lee et al. (5.2 μg/mg)^26^ and stands above the phospholipid-functionalized HNT systems described by Li et al. (73.4 μg/mg),^27^ demonstrating that the current platform provides a remarkably competitive drug capacity for therapeutic applications. That means the presence of the peptides does not affect the amount of DOX loaded.

Figure 4 shows the DOX release profiles from the two functionalized nanomaterials evaluated under both physiological (pH 7.4, Figure 4a) and acidic (pH 5.5, Figure 4b) conditions to mimic the healthy bloodstream and the tumor microenvironment/endosomal compartments, respectively.

**Figure 4:**
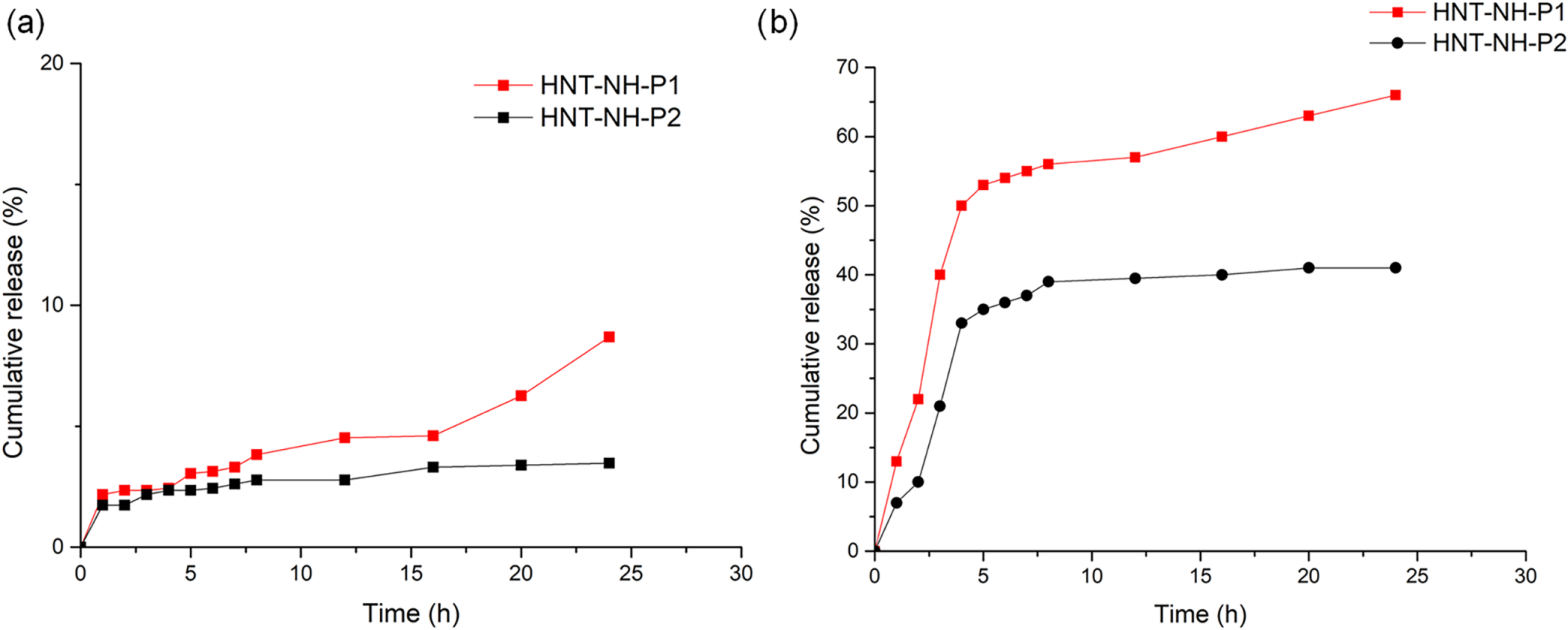
Drug release curves of DOX in HNT-NH-P1 (black line) and HNT-NH-P2 (red line): (a) at pH 7.4; (b) pH 5.5.

At pH 7.4, both systems exhibited a controlled and slow release behavior; during the first 5 h, they behaved similarly, releasing approximately 2.5% of the loaded DOX. Subsequently, the cumulative release from HNT-NH-P1 increased steadily up to 9% over 24 h, whereas HNT-NH-P2 showed a slower release kinetics, reaching only 3.5% within the same time frame.^16^ Conversely, a significant acceleration in the drug release kinetics was observed for both composites at pH 5.5 (Figure 4b). Under these acidic conditions, a pronounced initial burst release occurred within the first 5 h, with HNT-NH-P1 and HNT-NH-P2 releasing roughly 50 and 33% of their payload, respectively. By 24 h, the cumulative DOX release reached 66% for HNT-NH-P1 and plateaued at around 41% for HNT-NH-P2.^28^

### 4.3 Biological Activity

The effects of HNT, HNT-DOX, HNT-NH_2_, HNT-NH-P1-DOX, HNT-NH-P2-DOX, and free DOX on the viability and morphology of MCF-7 and MDA-MB-231 cells were evaluated at DOX concentrations of 2.5 and 5 μM, while, for HNT samples without DOX, equivalent amounts of nanocarrier corresponding to those used in the DOX-loaded formulations were used to maintain consistent carrier exposure. MTT assays showed that HNT and HNT-NH_2_ were noncytotoxic under both conditions in both cell lines, indicating that the nanocarrier system itself does not affect cell viability (Figure 5). In a previous experiment, the MDA-MB-231 cell line only (data not published), the free peptides were also tested and did not show relevant effects on cell viability; these data are now provided in the Supporting Information (Figure S8). In contrast, DOX-loaded nanocomposites (HNT-DOX, HNT-NH-P1-DOX, and HNT-NH-P2-DOX) induced a dose-dependent decrease in cell viability, although the cytotoxic effect was lower than that observed with free DOX, likely due to the limited availability of DOX from the carriers. At 5 μM, HNT-NH-P1-DOX appeared to be more effective in reducing cell viability compared to HNT-DOX and HNT-NH-P2-DOX, while no significant differences were observed between the two cell lines. Interestingly, despite the comparable DOX loading efficiencies measured for the different peptide-functionalized systems, HNT-NH-P1-DOX exhibited a greater cytotoxic effect, likely associated with its faster DOX release profile observed in the release studies.

**Figure 5:**
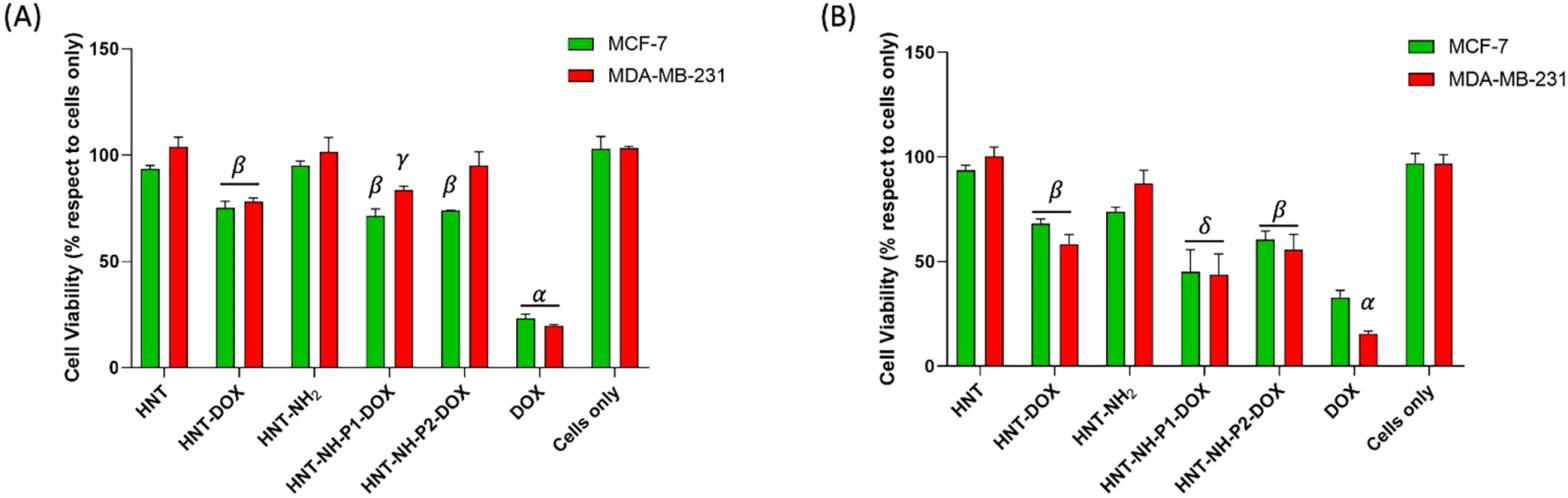
Cell viability analysis in MCF-7 and MDA-MB-231 cell lines. MTT assay was performed after 72 h of cell culture. The data show the percentage of viable cells compared to cells alone as the control (i.e., untreated cells), and the mean ± SEM is presented. The graphs show the viability of the cells at different concentrations of DOX: (A) 2.5 μM; (B) 5 μM. Statistical comparisons are summarized as follows: α (DOX effect): DOX vs all treatment groups (2.5 μM, *p* ≤ 0.0001; 5 μM, *p* ≤ 0.0001 except HNT-NH-P1-DOX in MCF-7 cells). β (carrier effect): untreated cells vs HNT-DOX and peptide-functionalized nanocarriers showing significant differences across both concentrations and cell lines as indicated in panels (A, B) (*p* ≤ 0.001). γ (P1-specific effect, low dose): untreated cells vs HNT-NH-P1-DOX in MDA-MB-231 cells at 2.5 μM (*p* ≤ 0.01). δ (P1-specific effect, high dose): untreated cells vs HNT-NH-P1-DOX at 5 μM in both cell lines (*p* ≤ 0.0001).

A qualitative analysis of cell morphology was also performed by fluorescent staining of actin filaments, supporting the observations from the MTT assays. At 5 μM, a clear reduction in cell density was observed in both MCF-7 and MDA-MB-231 cells, consistent with the decreased viability detected in the MTT assay (Figures 6 and S9). No evident differences in actin organization were observed between the two cell lines, suggesting that the cytotoxic effects of the composites are not cell line-specific.

**Figure 6:**
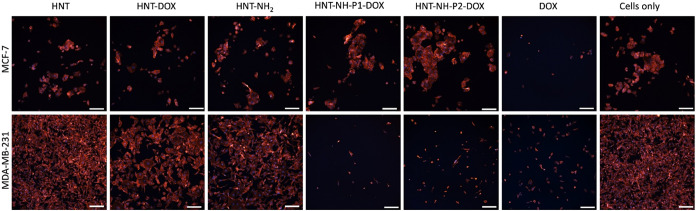
Cell morphology analysis. Fluorescent staining of actin filaments (in red) and cell nuclei (in blue) after 72 h of culture (DOX 5 μM). Scale bars: 200 μm.

To further investigate the potential of the HNT-based nanocarriers to inhibit cell migration, *in vitro* scratch assays were performed. Since HNT and HNT-NH_2_ were noncytotoxic and did not alter cell behavior, only DOX-loaded composites (HNT-DOX, HNT-NH-P1-DOX, and HNT-NH-P2-DOX) were tested. After 72 h, a marked inhibition of migration was observed in MDA-MB-231 cells for all DOX-loaded nanocomposites, which significantly inhibited migration in MDA-MB-231 cells (*p* ≤ 0.001). In contrast, in MCF-7 cells, a significant reduction in migration was detected only with HNT-NH-P2-DOX (*p* ≤ 0.05), while HNT-DOX and HNT-NH-P1-DOX had little to no effect (Figure 7).

**Figure 7:**
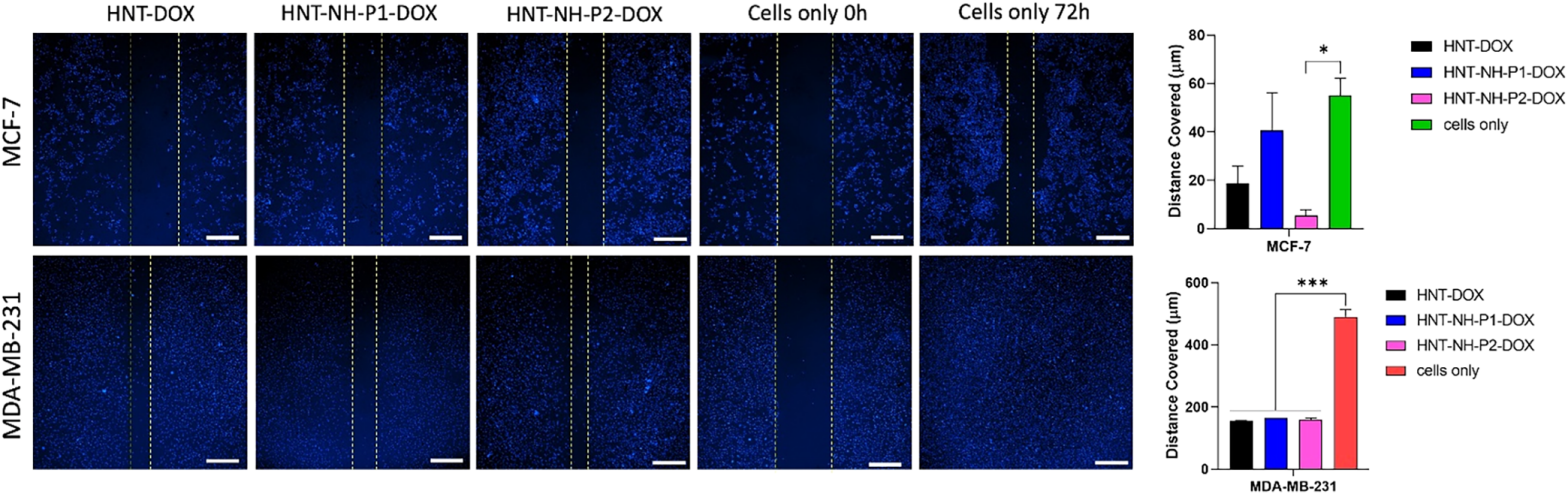
*In vitro* scratch assay. Representative images of DAPI-stained MCF-7 and MDA-MB-231 cells. Scale bars = 500 μm. Cell nuclei in blue. Yellow dotted lined defined the scratch. Quantitative analysis of scratch closure after 72 h. The scratch area was determined as the scratch area after 72 h relative to the original area. *p value ≤ 0.05, ***p value ≤ 0.001.

These preliminary results indicate that the two peptide-functionalized systems exert different effects depending on the specific cell lines. In MDA-MB-231 cells (c-Met-positive), all DOX-loaded formulations significantly reduced cell migration after 72 h. Conversely, in MCF-7 cells (c-Met-negative), HNT-NH-P1-DOX did not inhibit migration, unlike the other DOX-loaded systems. This selective behavior allows us to hypothesize that the antimigratory activity of HNT-NH-P1-DOX might be correlated with a c-Met-mediated interaction. Interestingly, despite the comparable DOX loading efficiencies measured for HNT-NH-P1-DOX and HNT-NH-P2-DOX, the two systems displayed different biological responses, suggesting that peptide functionalization mainly influences the cellular response and drug release behavior rather than the total amount of loaded drug. In particular, HNT-NH-P1-DOX, which exhibited the fastest DOX release profile, also showed the strongest reduction in cell viability, indicating a potentially greater drug availability at the cellular level. In contrast, HNT-NH-P2-DOX showed slower release kinetics but a more pronounced inhibition of cell migration, especially in MCF-7 cells, suggesting that the P2-functionalized system may preferentially affect mechanisms associated with cell motility and invasive behavior rather than acute cytotoxicity. These observations suggest that the nature of the peptide may contribute not only to receptor recognition but also to modulating the balance between cytotoxic and antimigratory effects. However, this remains a preliminary hypothesis; definitive confirmation of a specific receptor-targeted mechanism and the cellular internalization pathways will strictly require direct binding quantification and cellular uptake tracking in future investigations.

From a clinical perspective, restricting cancer cell migration and thus limiting the processes driving local invasion and distant metastasis is crucial, as metastatic dissemination accounts for the majority of cancer-related deaths. A nanocarrier capable of preferentially affecting c-Met-positive cells could reduce tumor spreading and metastatic potential.

Future studies should aim to validate these findings through targeted uptake analyses and *in vivo* models and to better elucidate the molecular basis of the observed c-Met-dependent selectivity.

Finally, our platform was implemented by adding an additional element to evaluate its potential suitability for future radiopharmaceutical applications. P1 was selected for further modification with a well-known chelating agent for Ga^3+^ such as THP (tris­(hydroxypyridinone)) with a NHS termination (THP-NHS).^15^ THP is widely used in PET diagnostics due to its ability to chelate the isotope ^68^Ga and other trivalent metals, such as Fe.^2,29–34^ Scheme 2 shows the synthetic strategy adopted for the functionalization of the HNT-NH-P1 composite with THP-NHS. In this case, the formation of an amide bond between the free NH_2_ groups of the composite and THP-NHS was exploited as a synthetic strategy, followed by complexation with GaCl_3_ in water.

**Scheme 2:**
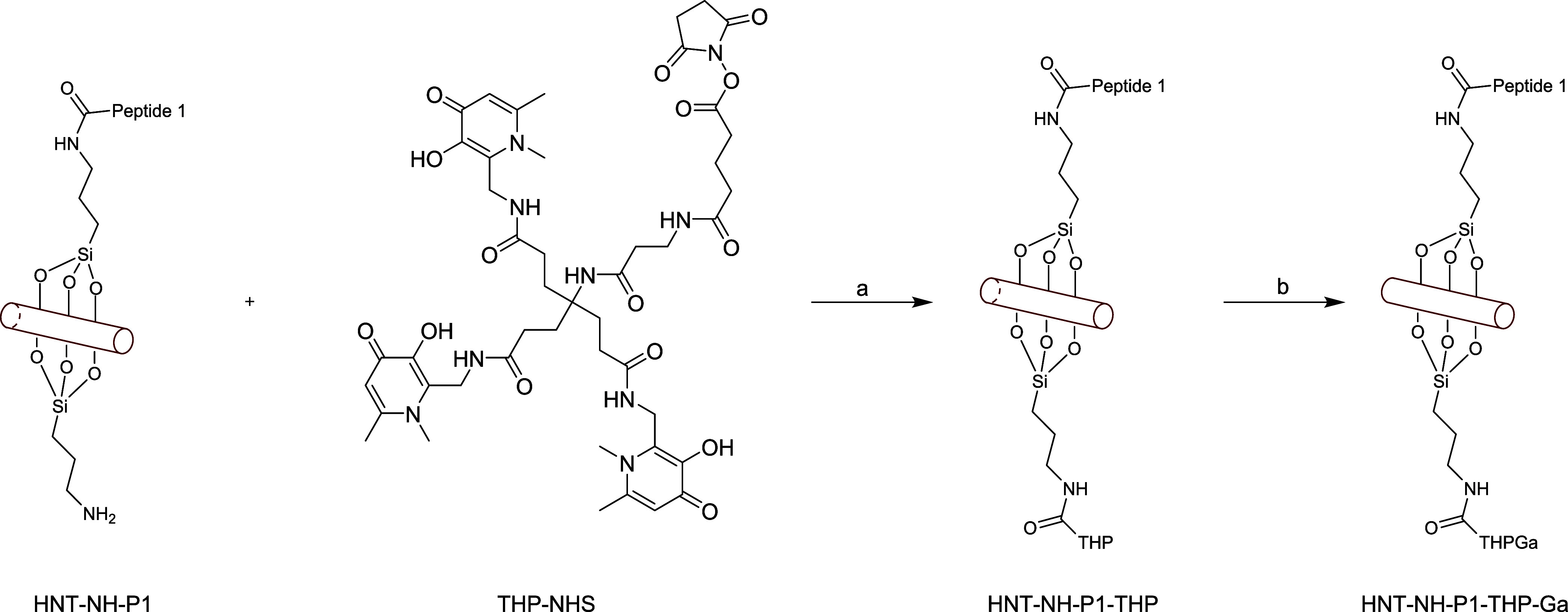
Preparation Scheme of HNT-NH-P1-THP-Ga; (a) Thp, r.t., 24 h; (b) GaCl_3_, H_2_O, r.t., o.v

The %*f* and the amount of Ga chelated by the composite were calculated using TGA, whose thermograms are shown in Figure 8.

**Figure 8:**
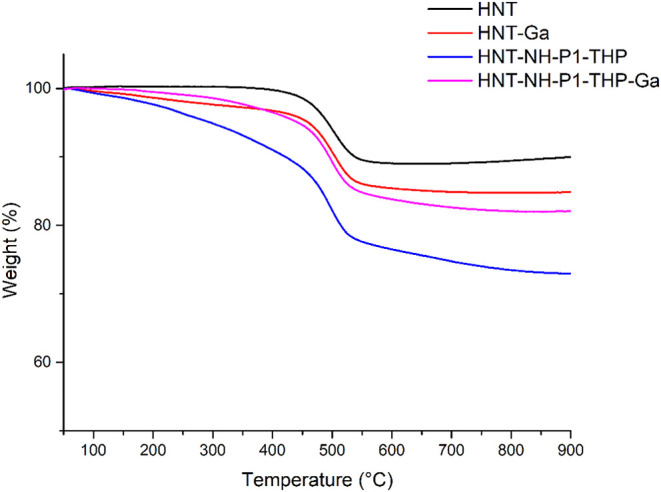
Thermogravimetric curves of HNT (black line), HNT-Ga (red line), HNT-NH-P1-THP (blue line), and HNT-NH-P1-THP-Ga (purple line).

From the thermograms, we can see that HNT-NH-P1-THP (Figure 8, blue line) has a residue of 73%, therefore a weight loss of 27%. Comparing this data with that of its precursor (HNT-NH-P1 22.46% weight loss, Table 1) shows an %*f* of approximately 4.5%. The amount of Ga chelated by THP was compared to unmodified HNT. Again, we calculated the amount of Ga from the thermograms by comparing the residues at 900 °C of HNT-Ga and HNT-NH-P1-THP-Ga (Figure 8, red and purple lines), which show a residue of 84.87 and 82.08%, respectively. These data, compared to the residues of the respective precursors (HNT and HNT-NH-P1-THP), allow us to say that HNT-Ga retains 1% of Ga and HNT-NH-P1-THP-Ga manages to chelate about 9% of Ga, proving the ability of THP to chelate this metal. While further radiochemical studies using active ^68^Ga, including radiolabeling efficiency, serum stability, and PET imaging, are strictly necessary to fully validate the theragnostic potential of this platform, this cold-chelation study serves as a fundamental chemical proof of concept.

## 5 Conclusions

This study successfully demonstrated the synthesis of a sustainable and ecologically biocompatible nanomaterial composed of halloysite nanotubes functionalized with targeting peptides (P1 and P2) for the delivery of doxorubicin. The use of tetrahydropyran as an environmentally friendly solvent throughout the synthesis highlights a commitment to sustainable chemical processes. Characterization confirmed the successful grafting of both the APTES linker and the two peptides onto the HNT surface. While the presence of the peptides did not affect the initial drug-loading efficiency, they had a significant impact on the subsequent release kinetics. The HNT-NH-P1 system showed a more rapid DOX release over 24 h compared to the HNT-NH-P2 system, suggesting that the specific peptide structure can be a key factor in modulating drug release rates. Biological assays highlighted a selective antimigratory behavior for the HNT-NH-P1-DOX system, allowing us to hypothesize a potential c-Met-mediated targeting mechanism. Although direct cellular uptake and binding affinity data are still required to fully validate this pathway, these findings are a crucial step toward creating advanced, targeted drug delivery systems that are both effective and environmentally conscious, opening doors for further research into optimizing peptide-based functionalization and exploring definitive molecular interactions for various therapeutic applications.

## Supplementary Material



## Notes

HPLC chromatogram of P1 (Figure S1); ESI mass spectrum of P1 (Figure S2); HPLC chromatogram of P2 (Figure S3); ESI mass spectrum of P2 (Figure S4); (a) UV spectra of DOX in PBS (pH: 7.4) at different concentrations; (b) calibration curve at 480 nm (Figure S5); energy dispersive X-ray (EDX) mapping analysis of HNT-NH-P1-DOX (Figure S6); energy dispersive X-ray (EDX) mapping analysis of HNT-NH-P2-DOX (Figure S7); cell morphology analysis. Fluorescent staining of actin filaments (in red) and cell nuclei (in blue) after 72 h of culture (DOX 2.5 μM). Scale bars: 200 μm (Figure S8) (PDF)
