# Supplementary material for: Anti-Invasive Peptide-Functionalized Nanotubes for Selective c‑Met Targeting and Metal Chelation
Source: ACS Omega. 2026 Jul 8;11(28):42706–15. doi: 10.1021/acsomega.6c03987 (PMC13393382; doi:10.1021/acsomega.6c03987)
Supplement: Supplementary file 1 [file ao6c03987_si_001.pdf]

Anti-Invasive Peptide-Functionalized Nanotubes for Selective C-Met Targeting and Metal Chelation

Vincenzo Patamia,[a] Noemi Ravaglia,[b,c] Mariacristina Failla,[d] Erika Saccullo,[a] Elena Bruno,[e] Vincenzo Abbate,[f] Monica Montesi,[b] Silvia Panseri,[b] Giuseppe Floresta,[a,*]

[a] *Department of Chemical Sciences, University of Catania, Viale Andrea Doria 6, 95125 Catania, Italy*

[b] *Institute of Science, Technology and Sustainability for Ceramics (ISSMC), National Research Council of Italy. Via Granarolo 64, 48018, Faenza, Italy.*

[c] *Department of Neuroscience, Imaging and Clinical Science, University of Studies "G. D'Annunzio", 66100 Chieti, Italy.*

[d] *Department of Drug Science and Technology, University of Turin, Via Pietro Giuria 9, 10125 Turin, Italy*

[e] *Department of Physics and Astronomy "Ettore Majorana", University of Catania, via S. Sofia 64, 95123 Catania, Italy. and CNR-IMM, University of Catania, Via S. Sofia 64, Catania Italy.*

[f] *Department of Analytical, Environmental & Forensic Sciences, Faculty of Life Sciences & Medicine, King's College London, London, UK*

**Corresponding authors*

e-mail: giuseppe.floresta@unict.it

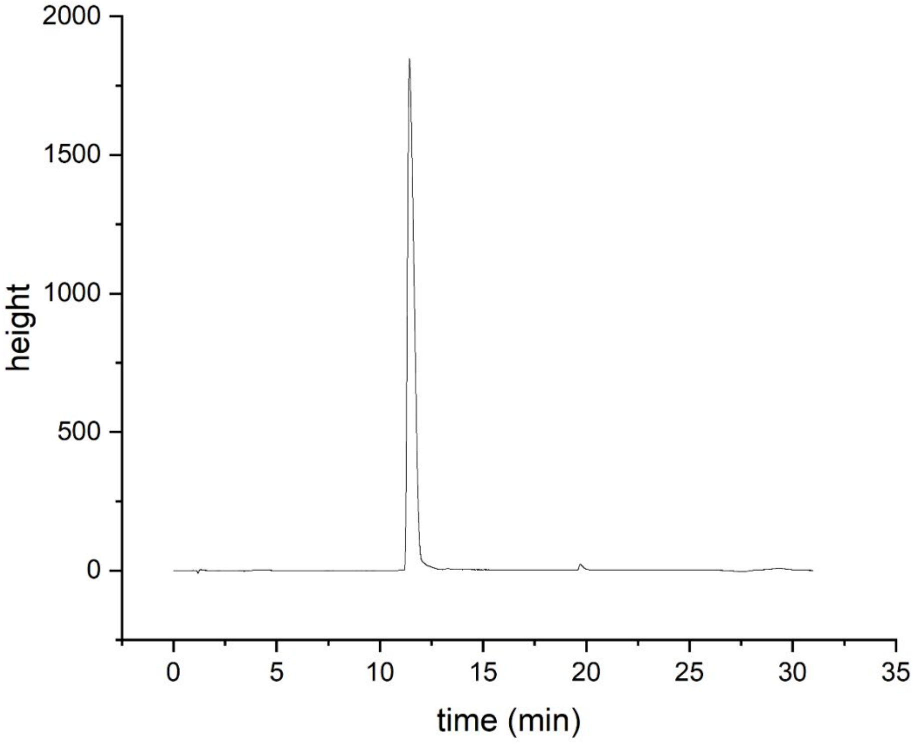

Figure S1. HPLC chromatogram of P1.

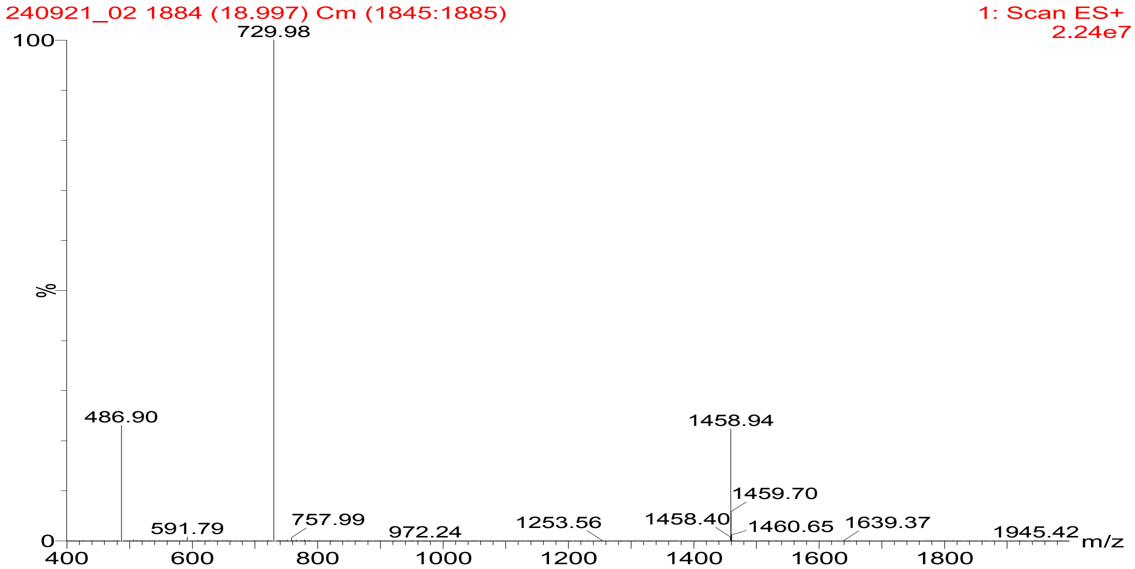

Figure S2. ESI mass spectrum of P1.

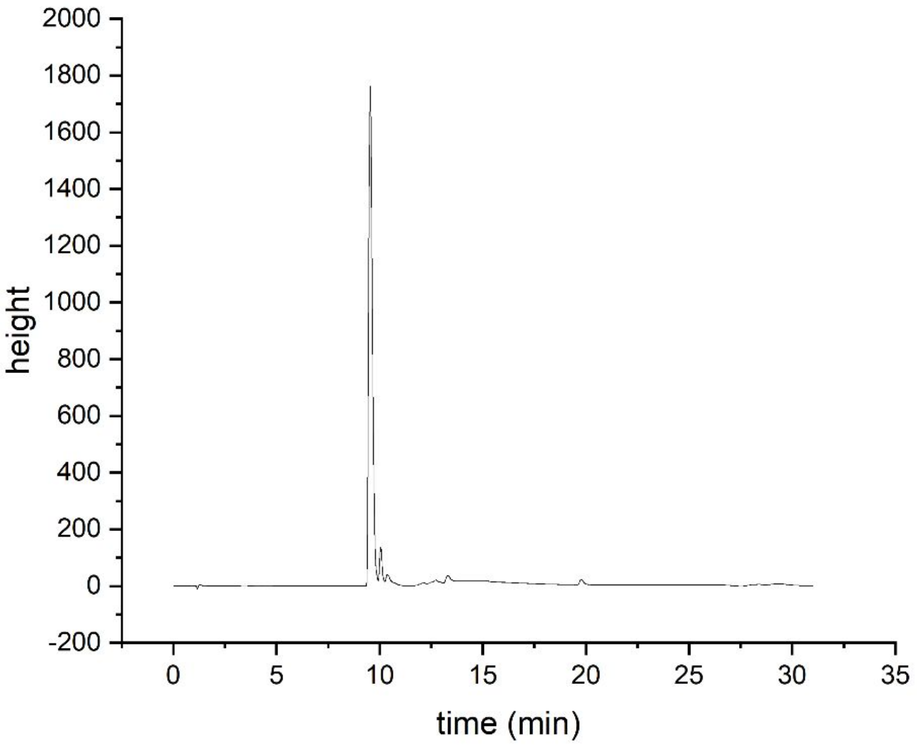

Figure S3. HPLC chromatogram of P2.

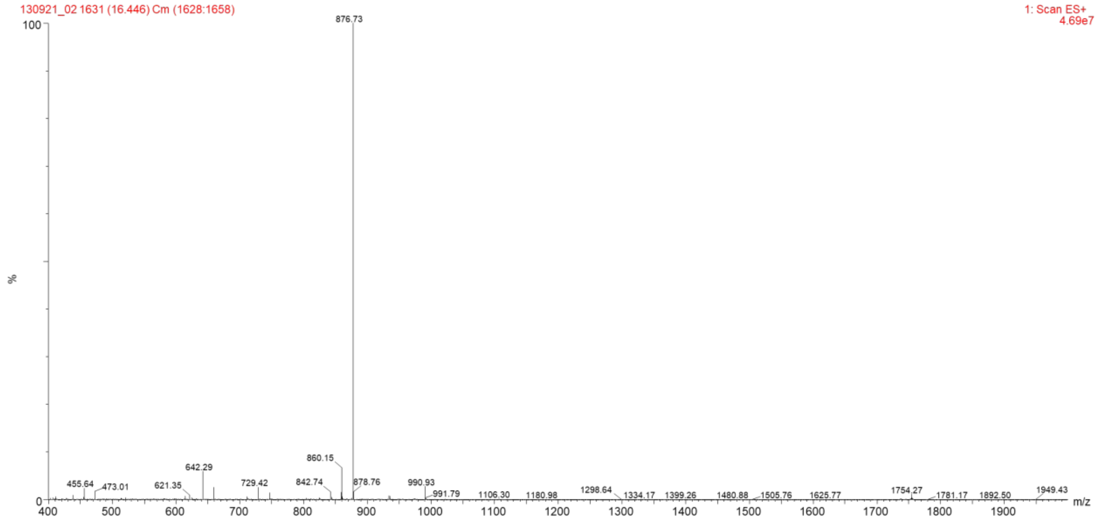

Figure S4. ESI mass spectrum of P2.

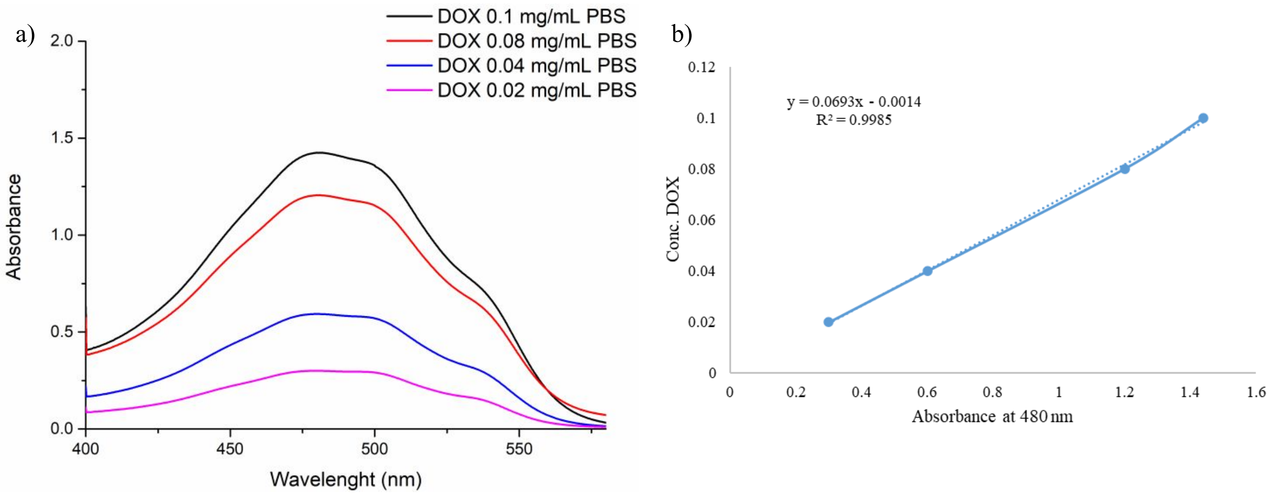

Figure S5. a) UV spectra of DOX in PBS (pH: 7.4) at different concentrations; b) calibration curve at 480 nm.

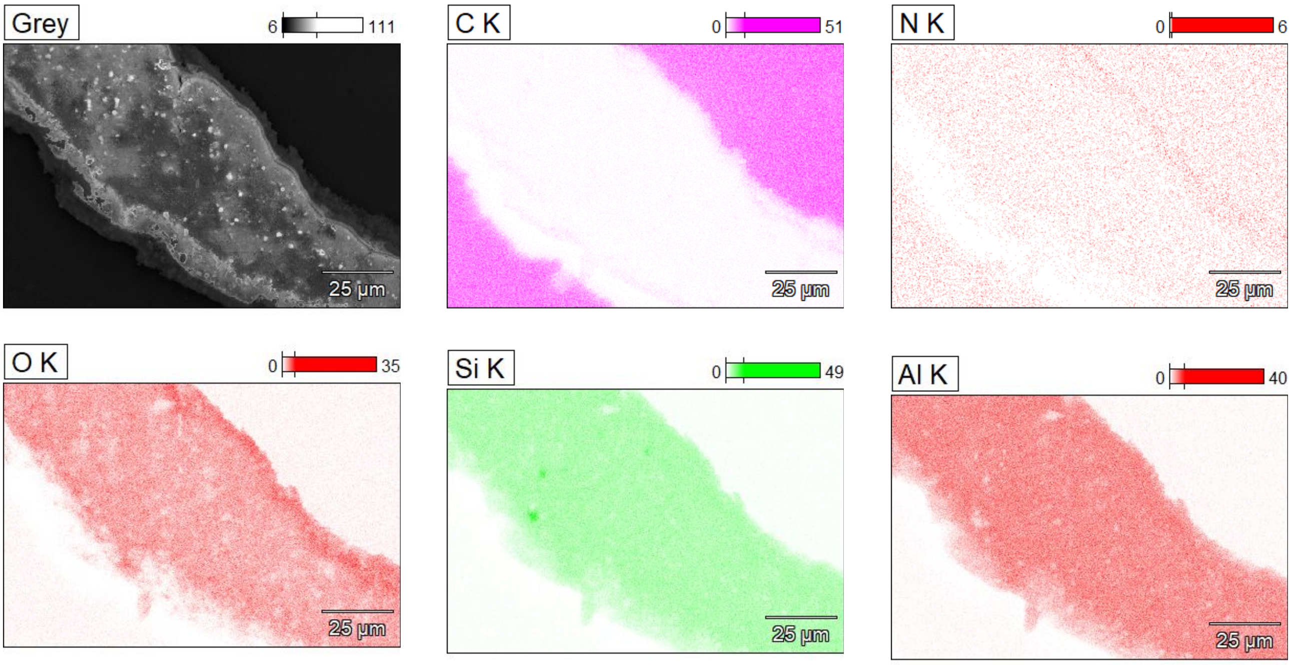

Figure S6. Energy dispersive X-ray (EDX) mapping analysis of HNT-NH-P1-DOX.

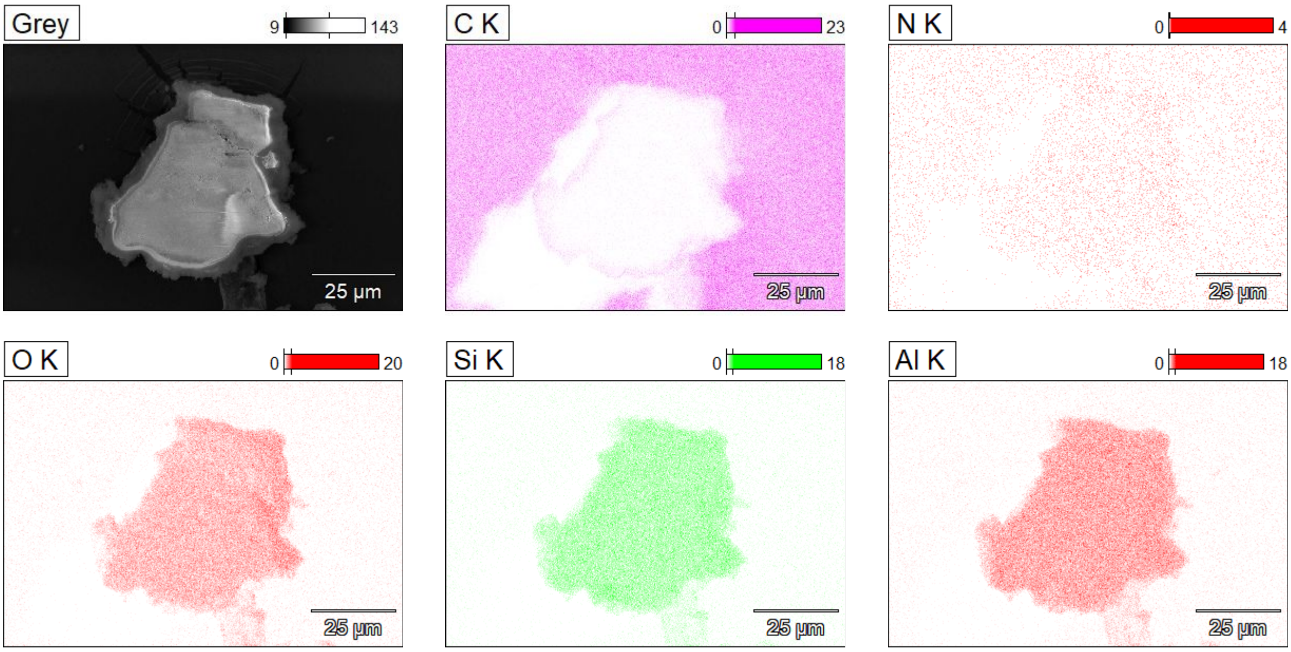

Figure S7. Energy dispersive X-ray (EDX) mapping analysis of HNT-NH-P2-DOX.

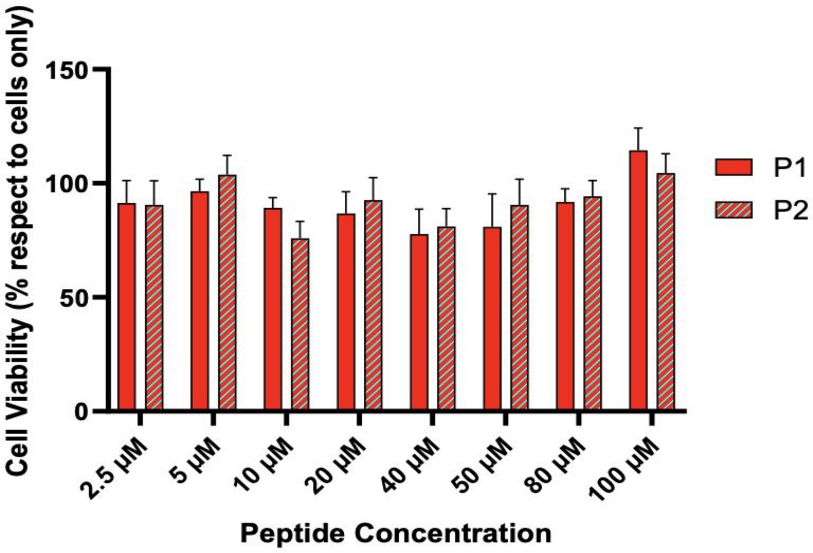

Figure S8. Cell viability analysis of the free peptides in MDA-MB-231 (c-Met positive) cell line. MTT assay was performed after 24 h of cell culture and 1 h incubation of the peptides at different concentrations. The data show the percentage of viable cells compared to cells alone as the control, and the mean ± SEM is presented.

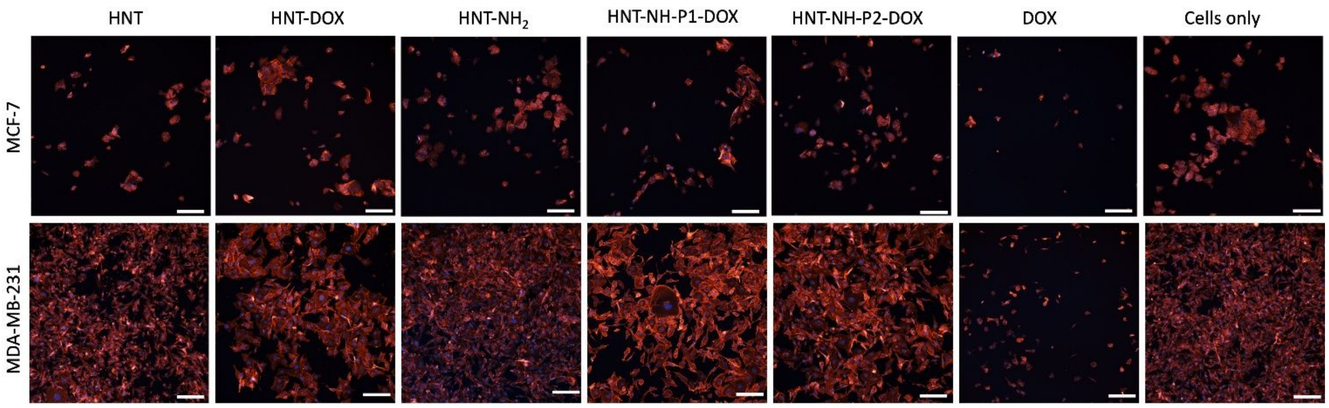

Figure S9. Cell morphology analysis. Fluorescent staining of actin filaments (in red) and cell nuclei (in blue) after 72h of culture (DOX 2.5 µM). Scale bars: 200 µm.
